# Targeting stromal cell Syndecan‐2 reduces breast tumour growth, metastasis and limits immune evasion

**DOI:** 10.1002/ijc.33383

**Published:** 2020-12-02

**Authors:** Paul G. Loftus, Luke Watson, Laura M. Deedigan, Eva Camarillo‐Retamosa, Róisín M. Dwyer, Lisa O'Flynn, Senthilkumar Alagesan, Matthew Griffin, Timothy O'Brien, Michael J. Kerin, Stephen J. Elliman, Laura R. Barkley

**Affiliations:** ^1^ Lambe Institute for Translational Research National University of Ireland Galway Ireland; ^2^ Orbsen Therapeutics National University of Ireland Galway Ireland; ^3^ Lisa O'Flynn, Avectas Ltd, Maynooth University Co Kildare Ireland

**Keywords:** breast cancer, Fc‐peptide, immunosuppression, syndecan‐2, TGFβ signalling, tumour‐associated stromal cells

## Abstract

Tumour stromal cells support tumourigenesis. We report that Syndecan‐2 (*SDC2*) is expressed on a nonepithelial, nonhaematopoietic, nonendothelial stromal cell population within breast cancer tissue. In vitro*,* syndecan‐2 modulated TGFβ signalling (*SMAD7, PAI‐1*), migration and immunosuppression of patient‐derived tumour‐associated stromal cells (TASCs). In an orthotopic immunocompromised breast cancer model, overexpression of syndecan‐2 in TASCs significantly enhanced TGFβ signalling (*SMAD7, PAI‐1*), tumour growth and metastasis, whereas reducing levels of *SDC2* in TASCs attenuated TGFβ signalling (*SMAD7, PAI‐1, CXCR4*), tumour growth and metastasis. To explore the potential for therapeutic application, a syndecan‐2‐peptide was generated that inhibited the migratory and immunosuppressive properties of TASCs in association with reduced expression of TGFβ‐regulated immunosuppressive genes, such as *CXCR4* and PD‐L1. Moreover, using an orthotopic syngeneic breast cancer model, overexpression of syndecan‐2‐peptide in TASCs reduced tumour growth and immunosuppression within the TME. These data provide evidence that targeting stromal syndecan‐2 within the TME inhibits tumour growth and metastasis due to decreased TGFβ signalling and increased immune control.

## Abstract 2

**What's new?**

Tumour‐associated stromal cells within the tumour microenvironment play a significant role in promoting tumorigenesis. Current strategies to inhibit tumour‐associated stromal cell function must however reduce the potential side‐effects associated with affecting normal stromal cells. In this study, the authors report that Syndecan‐2 is expressed on a non‐epithelial, non‐haematopoietic, non‐endothelial stromal cell population within breast cancer tissue. Syndecan‐2 contributes to the oncogenic properties of tumour‐associated stromal cells by promoting TGF‐β signalling, tumour growth, metastasis, and immunosuppression. Altogether, the results highlight pro‐tumorigenic, immunosuppressive Syndecan‐2+ stromal cells within the breast tumour microenvironment as a potential therapeutic target.

**Abbreviations**
BCCs: breast cancer cells
CAFs: cancer‐associated fibroblasts
CXCR4 C‐X‐C: chemokine receptor type 4
EMT: epithelial‐to‐mesenchymal transition
FAP: fibroblast activation protein alpha
FSP: fibroblast surface protein
MSC: mesenchymal stromal cells
NG2: neuron glia antigen‐2
PBMCs: peripheral blood mononuclear cells
PDGFR: platelet‐derived growth factor receptor
PD‐L1: programmed death ligand 1
Sdc2: Syndecan‐2
TASCs: tumour‐associated stromal cells
TBP: TATA‐box binding protein
TGFβ: transforming growth factor‐beta
TME: tumour microenvironment.
αSMA: alpha smooth muscle actin

## 1 INTRODUCTION

Although advances in therapeutic treatments and understanding the molecular pathways involved in breast cancer biology have been made, breast cancer is still a leading cause of cancer death in women.^1^ The tumour‐associated stroma has received increased attention for its role in initiating and sustaining tumour growth.^2^ The stroma is composed of heterogeneous cell types including; mesenchymal stromal cells (MSCs), cancer associated fibroblasts (CAFs), immune cells and endothelium.^3^ The importance of these cell types is indicated by the fact that a stroma‐related gene signature predicts resistance to chemotherapy in breast cancer.^4^ Therapies directed to cancer cells fail to eradicate stromal cells, which can re‐establish a tumourigenic environment and promote recurrence.^5^, ^6^ Studies indicate that fibroblast activation protein alpha (FAP)^+^, fibroblast surface protein (FSP)^+^, alpha smooth muscle actin (αSMA)^+^, CD45^−^ and CD11b^−^ stromal cells within lung and breast tumours promote tumourigenesis, creating an immunosuppressive niche within the tumour microenvironment (TME).^7^, ^8^, ^9^ An FAP^+^, platelet‐derived growth factor receptor (PDGFR)α^+^, PDGFRβ^+^, CD45^−^, EpCAM^−^, CD31^−^ CAF population isolated from mouse lung and melanoma tumours inhibit T‐cell function via programmed death ligands 1 and 2 (PD‐L1 and PD‐L2), which bind to the programmed death 1 receptor (PD‐1) on T cells.^10^, ^11^ In addition to FAP^+^, FSP^+^ stromal cells within ovarian carcinomas secrete factors that promote tumour growth by enhancing microvascularisation, stromal networks and protumourigenic paracrine signals.^12^ Studies indicate that MSCs secrete transforming growth factor‐β (TGFβ), and upregulation of the TGFβ signalling pathway in FAP^+^, EpCAM^−^, CD45^−^, CD31^−^ CAFs within human colorectal tumours predicts metastasis and defines a poor prognosis.^13^ TGFβ within the TME also promotes epithelial‐to‐mesenchymal transition (EMT), angiogenesis and mediates immunosuppression.^14^, ^15^, ^16^, ^17^ It is evident from these studies that it is difficult to distinguish between MSCs and CAFs within the TME as they express and secrete similar proteins. Nevertheless, discovering a regimen to target these tumour‐associated stromal cell (TASC) populations is a key goal in cancer medicine to reduce their protumourigenic influence on growth and metastasis, and remove their block on tumour immune recognition. Indeed, ablation of FAP^+^ TASCs reduced tumour size in mouse lung and pancreatic tumours due to increased immune control within the TME.^18^, ^19^ Additionally, ablation of tumour stromal neuron glia antigen‐2 (NG2)^+^ and PDGFRα^+^ pericytes significantly reduces breast tumour volume in preclinical studies.^20^ However, FAP^+^/NG2^+^/PDGFRα^+^ stromal cell depletion also causes side effects such as anaemia, cachexia and increased metastasis due to deletion of healthy stromal cells.^20^, ^21^, ^22^ These studies confirm that TASCs play a significant role in promoting tumour growth but also highlight that safer strategies are required to inhibit TASC function so as to reduce potential side effects due to effecting normal stromal cells.

Syndecan‐2 (*SDC2* (human) *Sdc2* (mouse)) is a heparan sulfate proteoglycan (HSPG) expressed in cells of mesenchymal origin.^23^, ^24^ Syndecan‐2 structure consists of a short cytoplasmic domain, a transmembrane domain and a larger extracellular domain, which is modified towards the N‐terminus with addition of heparan sulfate chains and glycosylation.^23^, ^25^ These specific functional domains enable syndecan‐2 to interact with cell membrane receptors, act as coreceptors for ligand binding, as well as activate signalling pathways that promote cell adhesion and migration.^26^, ^27^, ^28^ Syndecan‐2 expression is increased in cancers of breast, pancreas, colon and prostate.^29^, ^30^, ^31^, ^32^, ^33^, ^34^ In patients with ER‐negative breast cancer, high *SDC2* RNA expression in breast tumours correlates with poor prognosis.^31^ Additionally, inhibiting *SDC2* expression in MDA‐MB‐231 breast cancer cells (BCCs) reduced tumour volumes and improved survival in an adoptive transfer mouse model of breast cancer.^31^ Taken together, these studies indicate that epithelial syndecan‐2 can play a pro‐oncogenic role in breast cancer by promoting both tumour growth and migration. To date however, there have been no published investigations of syndecan‐2 expression or function within the stromal compartment of the breast TME. In this study, we report that syndecan‐2 is also expressed on the cell surface of a population of TASCs isolated from human and mouse breast tumours. Utilising in vitro and in vivo approaches, we find stromal syndecan‐2 has a key role in tumour growth, metastasis and immune evasion and is therapeutically targetable using a syndecan‐2‐derived peptide.

## 2 MATERIALS AND METHODS

### 2.1 Cell culture

Authenticated MDA‐MB‐231 (RRID:CVCL_0062) cells were obtained from American Type Culture Collections (Rockville, MD) in the last 3 years and maintained in Dulbecco's modified Eagle's medium (DMEM) supplemented with 10% fetal bovine serum (FBS), 100 U/mL penicillin and 100 mg/mL streptomycin at 37°C and 5% CO_2_. EO771 (RRID:CVCL_GR23) cells were obtained from Anderson et al^35^ and were maintained in DMEM supplemented with 10% FBS, 100 U/mL penicillin and 100 mg/mL streptomycin at 37°C and 5% CO_2_. Umbilical cord, bone marrow‐derived MSCs and human TASCs were maintained in α‐minimum essential medium (αMEM) with 10% FBS, 100 U/mL penicillin and 100 mg/mL streptomycin with 1 ng/mL human fibroblast growth factor 2. These were cultured at 37°C, 2% O_2_ and 5% CO_2_. Mouse TASCs were maintained in α‐MEM with 10% FBS, 10% equine serum, 100 U/mL penicillin and 100 mg/mL streptomycin. All experiments were performed with mycoplasma‐free cells.

### 2.2 Isolation of human TASCs

After ethical approval and written informed consent, fresh specimens of human breast tumours were harvested from patients undergoing surgery at University College Hospital Galway. Tissues were washed, minced finely and digested overnight with 0.1% collagenase type III at 37°C and 5% CO_2_. Collagenase‐dissociated mammary cells were pelleted at 400*g* for 5 minutes and cell pellets were resuspended in 2 mL of prewarmed trypsin‐EDTA by gentle pipetting and left to incubate at 37°C for 2 minutes. Trypsin was inactivated with Hanks' balanced salt solution supplemented with 2% FBS (HF). Cells were pelleted as before, resuspended in HF and filtered through a 100 μm cell strainer. Cells were pelleted and resuspended in FACS buffer (PBS [phosphate‐buffered saline] containing 2% FBS and 0.1% NaN_3_) or stromal cell growth medium and viable cells counted using a haemocytometer. A number of 100 000 cells were incubated for 30 minutes with CD45 or syndecan‐2 antibodies alone or in combination. Viability was assessed using Sytox blue staining. Data were collected using a BD FACS Canto II flow cytometer (BD Bioscience) and analysed using Flowjo software. Alternatively, cells were plated in TASC growth media and expanded as described earlier.

### 2.3 Tumour generation protocol

C57BL/6 mice were bred in‐house. NOD‐SCID mice were purchased from Charles River at 6 weeks of age, the mice were allowed 2 weeks to acclimatise. At 8 weeks of age, female mice were anaesthetised by isofluorane inhalation. A small incision was made just medial of the midline and lateral of the fourth nipple. EO771 or MDA‐MB‐231 BCC and TASC were injected at a ratio of 10:1 (1 × 10^6^ EO771:1 × 10^5^ TASC) (2 × 10^6^ MDA‐MB‐231:2 × 10^5^ TASC) in 100 μL of sterile PBS by pushing the needle proximally and parallel to the skin into the mammary fat pad before slowly injecting them. The needle was removed and the wound closed using Vetbond tissue adhesive (Medray). Tumour growth was monitored by calliper measurement. The long (L) and short (S) dimensions were taken and the approximate tumour volume calculated using the formula (L × S)^2^/2. Ethical and legal approvals were obtained prior to commencement of all animal experiments.

### 2.4 Tumour digestion and mouse TASC isolation

Upon excision, PyMT:ChOVA, EO771 and MDA‐MB‐231 tumours were roughly chopped using scissors and incubated at 37°C in digestion buffer (DMEM with collagenase 1 [1.5 mg/mL] and hyaluronidase [30 U/mL]) with periodic agitation. After 30 minutes, digestion buffer was removed and put in cold TASC media, fresh digestion buffer was added to cells for another 30 minutes at 37°C and this step was repeated once more. After the final digest, all remaining cells were added, combined and pelleted. The pellet was resuspended in 0.25% trypsin for 5 minutes before neutralisation using TASC media. Cells were pelleted and resuspended in ACK lysis buffer for 1 minute and neutralised with TASC media. Cells were resuspended in media and passed through a 100‐μm cell strainer to obtain a single cell suspension. Cells were counted using a NucleoCounter NC‐200 automated cell counter and plated at a density of 1.5 × 10^5^cells/cm^2^ for expansion. Alternatively, cells were resuspended in FACs buffer for flow cytometric analysis.

### 2.5 Determination of stromal cell content within mouse breast tumours by flow cytometry

Single cell suspensions were incubated with anti‐mouse CD16/32 blocking antibody (Biolegend) at 1/100 for 15 minutes on ice. For FAP staining, cells were incubated with sheep anti‐FAP antibody (R&D Systems) at 1/50 dilution for 30 minutes before washing and reblocking with CD16/32. Cells were then incubated with biotin at 1/1000 dilution for 30 minutes. After washing, cells were incubated with PE‐conjugated streptavidin at a 1/400 dilution for 30 minutes. Cells were then washed again before a 30‐minute incubation with a cocktail of anti‐Ter119 (BD Horizon), CD45 (BD Horizon), CD31 (BD Pharminogen), Sdc2 (R&D systems) and EpCAM (Biolegend) antibodies. Viability was assessed using Sytox blue staining. Data were collected using a BD FACS Canto II flow cytometer (BD Bioscience) and analysed using the Flowjo software.

Single cell suspensions from xenograft tumours first underwent mouse cell depletion, using a mouse cell depletion kit (Miltenyi Biotech). Cells were then washed and incubated with the following cocktail for 30 minutes EpCam 1/50 (Biolegend), gp38 1/100 (Biolegend), Syndecan‐2 1/50 (R&D Systems), PDL‐1 1/50 (eBioscience) and CXCR4 1/50 (eBioscience). Viability was assessed using Sytox blue staining. Data were collected using a MACSQuant Analyzer 10 flow cytometer (Miltenyi) and analysed using the Flowjo software.

### 2.6 Determination of immune cell content within mouse breast tumours by flow cytometry

After tumour digestion, single cell suspensions were washed and incubated with the following antibody cocktails. Cocktail 1: CD62L 1/200 (eBiosciences), CD4 1/200, CD8a 1/200, CD44 1/400 and CD25 1/200 (all from BD Biosciences). Cocktail 2: F4/80 1/100 (Biolegend), CD11c 1/50, Ly6C 1/50, Ly6G 1/100 and CD11b 1/50 (all from BD Biosciences). Cells were incubated with appropriate antibody cocktail for 30 minutes at 4°C in the dark. After incubation, cells were washed twice with FACS buffer and centrifuged at 400*g* for 3 minutes at 4°C to remove unbound Ab. Cells were then resuspended in 200 μL FACS buffer and analysed using a MACSQuant Analyzer 10 flow cytometer (Miltenyi Biotech). Sytox Blue viability stain was added immediately prior to acquisition. All analyses were carried out using the FlowJo software.

### 2.7 Haematoxylin and eosin staining of metastatic lesions in the lung

Lungs were catheterised, perfused with 10% neutral buffered formalin and sutured shut to maintain lungs in an inflated state. They were submerged in 10% formalin for 24 hours followed by 24 hours of 100% ethanol and 24 hours of 70% ethanol. Lungs were then processed using an Excelsior AS tissue processor and embedded in paraffin wax. Haematoxylin and eosin (H&E) staining was carried out using 6‐μm tissue sections according to standard procedures. Briefly, sections were deparaffinised with xylene, rehydrated in decreasing percentages of alcohol and washed with water. Rehydrated sections were stained in Mayer's haematoxylin solution for 6 minutes. Sections were washed in running tap water for 4 minutes to undergo “blueing”. Sections were counterstained with eosin for 2 minutes and rinsed in the water bath. Slides were dehydrated using a graded alcohol series ending with two changes of absolute alcohol. Slides were then cleared with xylene. Xylene was evaporated and sections were covered with Histomount xylene‐based mounting solution and slides were left in a 37°C oven overnight to set. Images were captured using a Leica bright‐field inverted microscope. Metastatic lesions were detected and each section was given a metastatic score: 1 = no visible metastatic lesion, 2 = 1 metastatic lesion, 3 = 2 metastatic lesions and 4 = 3 or more metastatic lesions.

### 2.8 Phenotypic analysis of TASCs

For staining, cells were washed with FACS buffer. Antibodies were diluted in 50 μL FACS buffer and added to the cells for 30 minutes at 4°C. After incubation, cells were washed twice with FACS buffer to remove unbound Ab. Cells were then resuspended in FACS buffer and analysed using a FACS Canto II (BD Biosciences). All analysis was performed using FlowJo. The monoclonal antibodies used were: gp38‐PE (e‐Biosciences), Syndecan‐2‐APC and NG2‐PE (R&D), CD105‐PE, MHCII‐FITC, CD29‐PeCy5, CD86‐AF488 (Biolegend), PDGFRα‐PE, CD14‐APC, CD34‐PE, CD15‐FITC, MHCI‐PeCy7, Cd73‐PE, CD90‐PE, CD45‐FITC, CD11b‐PeCy7, CD80‐PeCy7 and CD117‐PE (BD Biosciences).

### 2.9 Generation of *SDC2* fragment expression constructs and transfection

An IMAGE human cDNA clone for *SDC2* (Clone ID 6383) was obtained from Open Biosystems. To generate Fc‐tagged *SDC2* fragment, cDNA spanning the length of amino acid 1‐87 was engineered using PCR and the resulting DNA fragment was subcloned into *Eco* R1 and *Bgl* II sites within the multiple cloning site of the pFUSE‐hIgG1‐Fc1 expression plasmid (InvivoGen). All cloning was verified by sequencing (EuroFins).

Cell transfection was performed using FuGENE HD Nonliposomal Transfection Reagent (Roche) using manufacturers' guidelines. In brief, 500 ng of *SDC2‐Fc* vector or control empty vector were diluted in 100 μL of Opti‐MEM Reduced Serum Media and 3 μL of FuGENE HD transfection reagent was added. The transfection mixture was mixed by gentle pipetting and left to incubate for 10 minutes at room temperature. The transfection mixture was added dropwise to cells in a 6‐well plate.

### 2.10 siRNA transfection

TASCs were plated at 2 × 10^5^ cells per well in a 6‐well plate and allowed to adhere for 12 hours. A master mix was made per well containing: 7.5 μL TransIT‐X2 (Mirus) with 30 pmol of siRNA‐*SDC2*‐A (5′‐ GAG AAA CAC UCA GAC AGU CUG UUU A‐3′), siRNA‐*SDC2*‐B (5′‐ GCU UCA GGA GUG UAU CCU A‐3′) or siRNA control (Ambion 4390843) in 150 μL of OptiMEM and incubated at room temperature for 15 minutes. The transfection mix was added dropwise into each well and allowed to incubate for 48 hours.

### 2.11 Adenovirus construction and infection

Adenovirus construction was performed by Welgen Inc. Recombinant adenovirus expressing full length *SDC2* (Ad*SDC2*) or a short hairpin RNA to SDC2 (sh*SDC2*) was generated. shRNA targeting *SDC2* (5′‐ GAG AAA CAC UCA GAC AGU CUG UUU A‐3′). Cells were routinely infected with adenovirus at a multiplicity of infection of 375. To control for adenoviral infection, cells received AdCon (“empty” adenovirus vector) or shCon (nontargeting shRNA control virus).

### 2.12 TGFβ treatment

Cells were plated in 6‐well plates at a density of 2.2 × 10^4^ cells/cm^2^. 12 hours later, cells were transduced or transfected and left to incubate for 48 hours. Cells were incubated in serum‐free medium for 12 hours, and TGFβ3 (R&D) was added at a concentration of 20 ng/mL for stromal cells or 5 ng/mL for MDA‐MB‐231 cells. Cells were harvested at 0 and 2 hour timepoints, by first washing twice with PBS followed by addition of 350 μL RLT lysis buffer (Qiagen).

### 2.13 Cumulative population doublings

TASCs were seeded into T75 flasks at a concentration of 1 × 10^5^ cells per flask and allowed to grow for 7 days with media change every 2 to 3 days. After 7 days, cells were trypsinised and counted and 1 × 10^5^ cells were reseeded into a T75 flask and allowed to grow for 7 days as before. This was repeated for a total of three passages and cumulative population doubling was calculated.

### 2.14 Cell viability

Cell enumeration and viability were measured with a ChemoMetec Nucleocounter NC‐200, Via1‐Cassette. 60 μL of a cell suspension was drawn into a Via1‐Cassette, which contained two immobilised fluorophores acridine orange (AO) and DAPI. AO stained all cells in the sample giving total cell number, DAPI‐stained dead cells. Via1‐Cassette was inserted into the ChemoMetec Nucleocounter NC‐200 and a viability and cell count protocol initiated.

### 2.15 RT‐qPCR analysis

Total RNA was isolated using the RNeasy mini kit (Qiagen) and cDNA synthesised using a high capacity cDNA reverse transcription kit (Applied Biosystems) following manufacturers guidelines. mRNA analyses were performed using TaqMan Gene Expression Assays (Applied Biosystems). Relative quantification was performed using TATA‐box binding Protein (TBP) as endogenous control.

### 2.16 Immunoblotting

Cells were lysed using 50 mM Tris buffer pH 7.4, 300 mM NaCl, 1 mM EDTA and 1% Triton‐X‐100 containing protease and phosphatase inhibitors. The resulting samples were normalised for protein content, then separated by SDS‐PAGE, transferred to nitrocellulose and analysed by immunoblotting with the following antibodies: human Fc antibody (R&D Systems), anti‐PD‐L1 (Invitrogen Molecular Probes, Eugene, OR) and anti‐Actin (Santa Cruz Biotechnology).

### 2.17 Colony formation assay

Cells were plated in a 6‐well plate and transfected with 500 ng of *SDC2* fragment ‐Fc DNA or empty vector control using FuGENE HD transfection reagent. 48 h later, cells were trypsinised, counted and replated at a density of 500 cells/10‐cm dish. 10 days later, colonies resulting from the surviving cells were fixed, stained with crystal violet and counted.

### 2.18 Migration assay

xCELLigence assays were set up following manufacturer's instructions. In brief, 160 μL of test medium was added to the bottom half of an RTCA DP CIM‐Plate 16, ensuring there were no air bubbles. The top half of the plate was fixed to the bottom and 50 μL of serum‐free medium was added to the wells. Cells were prepared and washed three times in serum‐free medium, cells were resuspended at 4 × 10^5^ cells/mL, 100 μL of this cell suspension was added per test well, the plate was incubated at 37°C and 5% CO_2_ for 30 minutes. CIM plates were inserted into the xCelligence plate and cell migration was measured every 15 minutes over a 48 hour period.

### 2.19 T‐cell proliferation assay

Peripheral blood mononuclear cells (PBMCs) were isolated from whole human blood using Ficoll (Fisher) gradient centrifugation. Cell suspensions were washed in PBS and stained with 10 μM carboxyfluorescein diacetate succinimidyl ester (CFSE) cell stain (CellTrace CFSE Cell Proliferation Kit, Invitrogen). Cells were incubated for 6 minutes at 37°C protected from light, reaction was stopped by adding an excess of ice‐cold T‐cell media (10% FBS, 2 mM Pen/Strep, 2 mM l‐glutamine, 0.1 M nonessential amino acids, 1 mM sodium pyruvate and 55 μM β‐mercaptoethanol in RPMI 1690 [Sigma Aldrich]). 2 × 10^5^ CFSE‐labelled T cells were added to a 96‐well plate, they were stimulated using mouse anti‐human CD3 0.5 mg/mL (BD Biosciences) and mouse anti‐human CD28 10 mg/mL (BD Biosciences). Stromal cells were added at ratios of 1:10, 1:50 and 1:200. Cells were harvested after 4 days and stained using PE‐Cy7 mouse anti‐human CD4 (BD Biosciences) and analysed using a FACS Canto II (BD Biosciences). All analyses were carried out using FlowJo. ≥3 generations of CD4^+^ T‐cell proliferation was determined in order to quantify the ability of stromal cells to suppress sustained proliferation.

### 2.20 Statistical analysis

Experiments were carried out 3 times. Values are presented as the mean ± SEM. Data were compared by two‐way analysis of variance (ANOVA) unless otherwise stated.

## 3 RESULTS

### 3.1 Syndecan‐2 is a cell surface stromal cell marker within breast tumours

**FIGURE 1:**
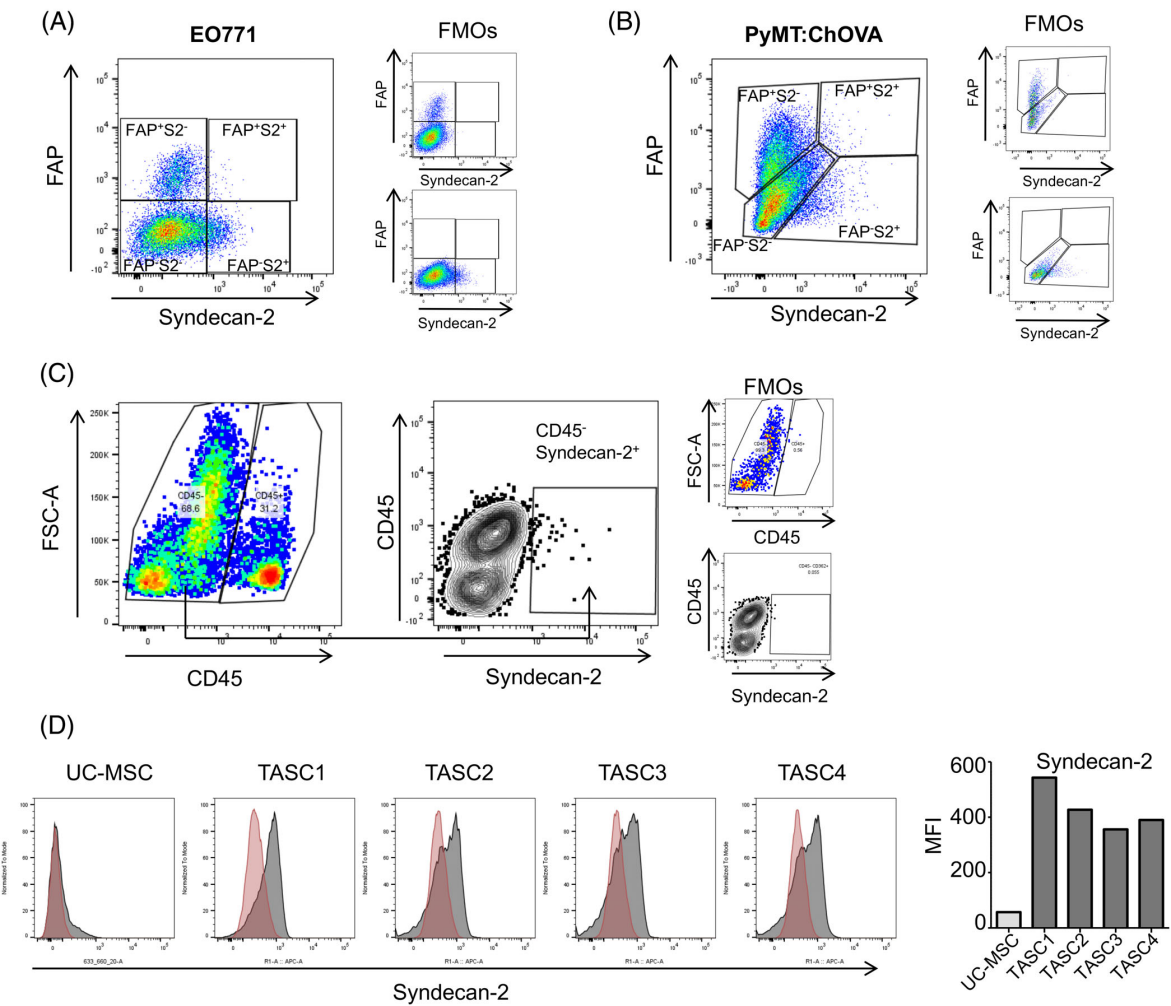
Syndecan‐2^+^ stromal cells within breast tumours. A, EO771 tumours were dissected and single cell suspensions stained with Syndecan‐2, FAP, CD45, Ter119, CD31 and EpCAM antibodies. TASCs were defined by flow cytometry as being CD45^−^ (nonlymphocyte) Ter119^−^ (nonerythroid), EpCAM^−^ (nonepithelial) and CD31^−^ (nonendothelial) cells. The levels of viable (Sytox ‐ve) Syndecan‐2^+^ stromal (CD45^−^Ter199^−^CD31^−^EpCAM^−^) were determined. Fluorescence minus one (FMO) controls were used to identify and gate cells. B, 24‐week‐old PyMT:ChOVA spontaneous tumours were dissected and single cell suspensions stained as earlier. C, Human breast cancer samples were digested and single cell suspensions stained with Syndecan‐2 and CD45 antibodies to determine the levels of viable (Sytox ‐ve) Syndecan‐2^+^/CD45^−^ and Syndecan‐2^+^/CD45^+^ cells by flow cytometry. D, Flow cytometry histograms and mean fluorescence intensity (MFI) illustrating cell surface expression of Syndecan‐2 [Color figure can be viewed at wileyonlinelibrary.com]

Syndecan‐2 has been shown to be highly expressed in cells of mesenchymal origin^23^, ^24^; therefore, we investigated Syndecan‐2 expression in the stromal compartment of breast tumours. Syngeneic EO771^35^ and spontaneous PyMT:ChOVA^36^ mouse breast tumours were isolated, digested and syndecan‐2 surface expression was analysed. To determine TASC expression of syndecan‐2 and compare it with expression of an alternative TASC marker, costaining for syndecan‐2 and FAP was examined within the stromal compartment. This identified four different stromal subpopulations, syndecan‐2^+^FAP^−^, syndecan‐2^+^FAP^+^, syndecan‐2^−^FAP^+^ and syndecan‐2^−^FAP^−^ (Figure 1A,B). Approximately 2% of TASCs isolated from EO771 tumours expressed syndecan‐2, 1.94 ± 0.35% syndecan‐2^+^FAP^−^ and 0.02 ± 0.008% syndecan‐2^+^FAP^+^ (mean ± SEM, n = 3 tumours from 3 different mice). 4% of TASCs isolated from PyMT:ChOVA tumours express syndecan‐2, 3.88% syndecan‐2^+^FAP^−^ and 0.5% syndecan‐2^+^FAP^+^. These data suggest that syndecan‐2 is expressed on the cell surface of a proportion of TASCs within two different mouse breast cancer models.

To determine if syndecan‐2 was also expressed in TASCs within human breast tumours, samples of primary tumours obtained during resection surgeries were digested and stained with antibodies against CD45 and syndecan‐2 and then analysed by flow cytometry. A syndecan‐2^+^CD45^−^ population of cells that represented ~1.955 ± 1.3% (mean ± SEM, n = 3) of the total tumour cells was identified (Figure 1C). In this case, syndecan‐2^+^CD45^−^ cells could represent syndecan‐2^+^ epithelial and/or stromal cells. Therefore, to further establish the presence of syndecan‐2^+^ TASCs, human breast tumours were digested and single cell suspensions grown under conditions that encouraged only stromal cell adherence and expansion. Using this method, we were able to isolate a population of adherent cells from breast cancer tissue from four patients with luminal breast cancer. Morphologically, these TASCs were similar to MSCs isolated from bone marrow (BM‐MSCs) and umbilical cord (UC‐MSCs) tissue (Supplementary Figure 1A). Patient‐derived TASCs expressed stromal markers CD90, CD105 and CD73 (Supplementary Figure 1B). Cells were negative for haematopoietic markers CD45 and CD34, monocyte/macrophage markers CD14 and CD11b, and lack MHC‐II expression, further confirming these cells are stromal cells (Supplementary Figure 1B).^37^ Additionally, cells were positive for the expression of known tumour stromal cell markers NG2, PDGFRα and podoplanin (gp38) (Supplementary Figure 1C).^38^ Flow cytometry analysis demonstrates patient‐derived culture‐expanded TASCs express cell surface syndecan‐2 to a greater extent compared to normal culture‐expanded UC‐MSCs (Figure 1D). Similar to mouse breast tumours (Figure 1A,B), syndecan‐2 is expressed on the cell surface of a proportion of human patient‐derived TASCs, further implying that TASCs are a heterogeneous population of cells within mouse and human tumours. Taken together, these results indicate that a NG2^+^PDGFRα^+^gp38^+^ stromal cell population isolated from primary human breast tumours are positive and negative for markers that define stromal cell lineage and a subpopulation of TASCs express syndecan‐2.

### 3.2 Syndecan‐2 regulates TGFβ signalling, migration and immunosuppressive properties of TASCs

**FIGURE 2:**
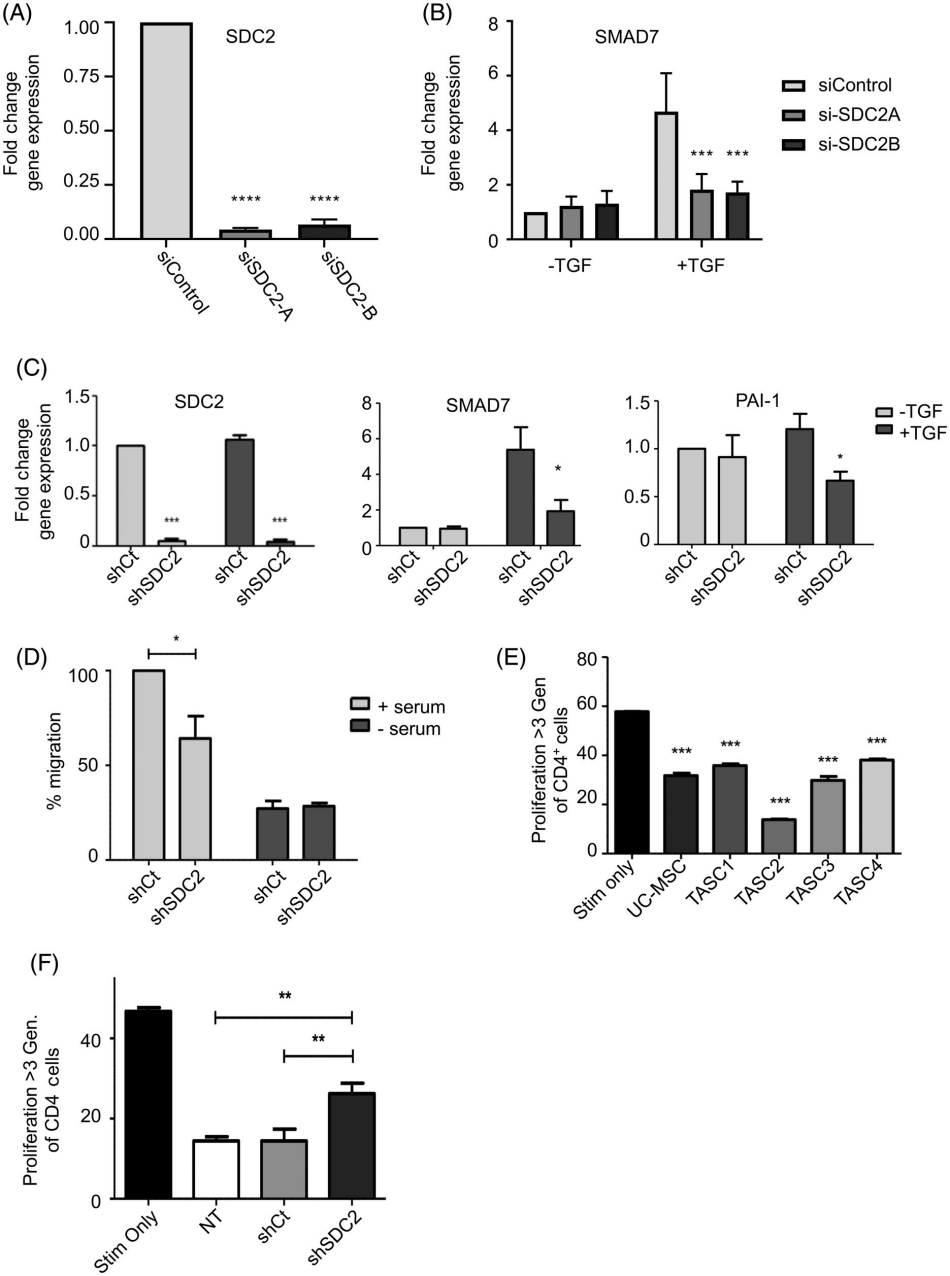
Modulation of *SDC2* effects TGFβ signalling, migration and immunosuppressive properties of TASCs. A, RT‐qPCR analysis showing siRNA‐*SDC2* transfected TASCs (si*SDC2*‐A and si*SDC2*‐B) have reduced *SDC2* RNA levels compared to siControl transfected TASCs. B, siControl or si*SDC2* transfected TASCs were treated with TGFβ. 2 hours after TGFβ treatment RNA was prepared and levels of *SMAD7* were determined by RT‐qPCR. n = 3; ***P ≤ .0001. C, shControl (shCt) or sh*SDC2* transduced TASCs were treated with TGFβ. 2 hours after TGFβ treatment RNA was prepared and levels of *SDC2* and TGFβ‐regulated genes were determined by RT‐qPCR. n = 4; *P ≤ .05 ***P ≤ .0001. D, The xCelligence system was used to determine the ability of shCt and sh*SDC2* transduced TASCs to migrate towards serum‐containing media. Levels of migration were normalised to the positive control (ie, shCt +serum). n = 4 *P ≤ .05. E, TASCs and umbilical cord MSCs (UC‐MSCs) were cocultured with CD3/CD28‐activated peripheral blood mononuclear cells (PBMCs) at a 1:50 ratio. Flow cytometry of CFSE‐labelled CD4^+^ T cells reveal CD3/CD28‐mediated proliferation is inhibited by TASCs. One‐way analysis of variance (ANOVA) with Tukey's multiple comparison test ***P ≤ .001. F, shCt or sh*SDC2* transduced TASCs were cocultured with CD3/CD28‐activated peripheral blood mononuclear cells (PBMCs) at a ratio of 1:50. Flow cytometry of CFSE‐labelled CD4^+^ T cells was used to measure the level of proliferation. Data were compared by one‐way ANOVA with Tukey's multiple comparison posttest. n = 4 (**P ≤ .01)

To date, there are no studies reporting the presence of syndecan‐2^+^ TASCs within the breast TME or the effect of manipulating syndecan‐2 within the stromal compartment of tumours, therefore we wanted to establish the role of this syndecan‐2^+^ TASC population within the TME. To determine this, we tested the effectiveness of two siRNA *SDC2* duplexes (si*SDC2*‐A and si*SDC2*‐B) to reduce *SDC2* levels in TASCs. Figure 2A indicates that both siRNAs effectively reduced *SDC2* RNA levels. It has been previously shown that syndecan‐2 is required for efficient TGFβ signalling,^28^, ^39^ therefore we examined if knockdown of *SDC2* affects TGFβ signalling in TASCs. Figure 2B shows that TGFβ induces expression of *SMAD7* in siRNA control TASCs, and this is reduced in si*SDC2*‐A and si*SDC2*‐B expressing TASCs. The sequence of si*SDC2*‐A was used to generate an shRNA‐*SDC2* adenovirus for subsequent experiments. To ensure that transduction of TASCs with adenovirus shControl or adenovirus sh*SDC2* did not alter the phenotype of primary TASCs, flow cytometry analysis was used to verify expression of stromal cell markers CD90, CD105 and CD73 (Supplementary Figure 2). After transduction, cells were also negative for haematopoietic markers CD45 and CD34, monocyte/macrophage markers CD14 and CD11b, and lack MHC‐II expression. This confirmed viral transduction did not alter stromal cell markers or induce expression of other cell markers. It was important to ensure shRNA‐mediated knockdown of *SDC2* (sh*SDC2*) also caused a reduction in TGFβ signalling. Loss of *SDC2* in sh*SDC2*‐transduced TASCs causes a reduction in TGFβ‐regulated factors—*Smad7* and *PAI‐1*—in comparison with shControl TASCs (Figure 2C). This further confirms that syndecan‐2 is important for efficient activation of the TGFβ pathway in TASCs.

Syndecan‐2 affects the migratory properties of breast cancer epithelial cells^31^ and TGFβ controls the migratory properties of MSCs,^14^, ^15^ therefore we wanted to ascertain if modulation of syndecan‐2 affected the migratory properties of TASCs. In TASCs, loss of *SDC2* causes a reduction in the ability of TASCs to migrate towards serum‐containing medium (Figure 2D).

A significant role of TASCs within the TME is immunosuppression.^7^, ^8^, ^9^, ^18^, ^19^ Consequently it was important to determine if TASCs isolated from human primary breast tumours possess immunosuppressive properties. MSCs isolated from umbilical cord (UC‐MSC) suppress CD4^+^ T‐cell proliferation,^40^ therefore T‐cell proliferation assays were performed whereby CD4^+^ T‐cell proliferation was quantified in the absence or presence of TASCs. Figure 2E indicates that incubating TASCs isolated from four different primary breast tumours with peripheral blood mononuclear cells (PBMCs) at a ratio of 1:50 suppressed CD4^+^ T‐cell proliferation. The mean level of suppression was 45.0% ± 1.7%, which was comparable to that mediated by UC‐MSC, 49.1 ± 5.0% (Figure 2E). TGFβ controls the immunosuppressive properties of MSCs^17^ and as syndecan‐2 affects TGFβ signalling, we wanted to determine if modulation of syndecan‐2 affected the immunosuppressive properties of TASCs. Therefore, the ability of sh*SDC2*‐transduced TASCs to suppress CD4^+^ T‐cell proliferation was compared to shControl‐transduced TASCs. shControl‐transduced TASCs reduced CD4^+^ T cell by 69.2 ÷ 6.4% (n = 4) compared to control CD3/CD28‐activated T cells, which is comparable to the level of suppression observed with nontransduced TASCs (69.1 ± 2.3%; n = 4; Figure 2F). TASCs with decreased levels of *SDC2*, however, are less effective at suppressing CD4^+^ T‐cell proliferation (43.9 ± 5.6% [n = 4]) compared to control cells (69.2 ± 6.4% [n = 4]).

Taken together, these data suggest that syndecan‐2 is important for TGFβ signalling and impacts both the migratory and immunosuppressive properties of patient‐derived TASCs.

### 3.3 Manipulation of *SDC2* in TASCs within breast tumours in vivo

**FIGURE 3:**
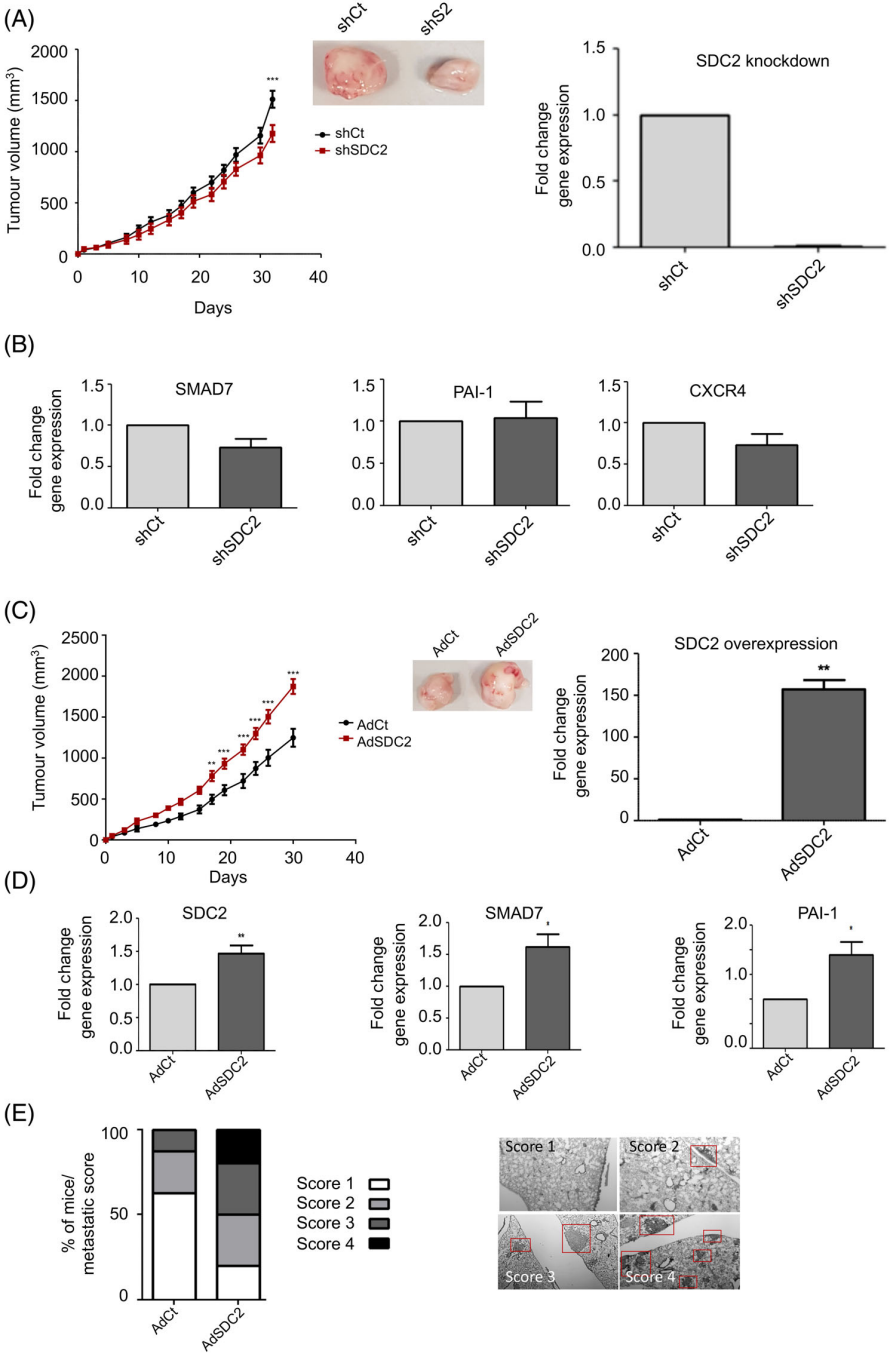
Manipulation of *SDC2* within the stromal cell compartment of xenograft tumours effects breast carcinogenesis. A, Orthotopic xenograft tumours were established by coinjecting MDA‐MB‐231 with sh*SDC2*‐transduced TASCs or shCt‐transduced TASCs into the mammary fat pad of immune‐compromised mice at a ratio of 1:10 (TASCs:MDA). Tumours containing sh*SDC2*‐TASCs (n = 9) showed significantly lower growth rates when compared to control shCt‐TASC tumours (n = 10). RT‐qPCR analysis showing sh*SDC2*‐transduced TASCs have reduced *SDC2* RNA levels compared to adenovirus shCt‐transduced TASCs. B, RNA was prepared from xenograft tumours and RT‐qPCR was performed to compare the expression of TGFβ‐regulated genes between shCt and sh*SDC2* expressing tumours (n = 5/group). C, Orthotopic xenograft tumours were established as described earlier. Tumours containing TASCs overexpressing *SDC2* (Ad*SDC2*) (n = 10) showed significantly higher growth rates when compared to control tumours (AdCt) (n = 9). RT‐qPCR analysis showing Ad*SDC2*‐transduced TASCs have increased *SDC2* RNA levels compared to adenovirus AdCt‐transduced TASCs. D, RNA was prepared from xenograft tumours and RT‐qPCR was performed to determine the effect of *SDC2* modulation within TASCs upon the expression of TGFβ‐regulated genes. (n = 5/group). E, Approximately 12 weeks after TASC:MDA injection, lungs were removed and examined for metastatic nodules by H&E staining. (AdCt [n = 9], Ad*SDC2* [n = 10]). The bar graph indicates the metastatic score/lung from the various experimental groups. Red boxes indicate metastatic lesions. *P ≤ .05; **P ≤ .01; ***P ≤ .001 [Color figure can be viewed at wileyonlinelibrary.com]

To ascertain if syndecan‐2 within the stromal compartment of the TME had a role in breast carcinogenesis in vivo, an orthotopic breast cancer model was established whereby MDA‐MB‐231 BCCs were coinjected with patient‐derived TASCs at a ratio of 10:1 into the mammary fat pad of NOD‐SCID mice. The effect of *SDC2* knock down in TASCs, by transduction with sh*SDC2*, on tumour growth was compared to that of tumours generated with shControl‐transduced TASCs (Figure 3A). Reduced levels of *SDC2* in TASCs was associated with a significant decrease in tumour growth compared to that of shControl tumours and reduced levels of TGFβ‐regulated genes, for example, *SMAD7* (Figure 3B **)**. Although there was no observed difference in *PAI‐1* expression between sh*SDC2* and shControl tumours, there was a decrease in *CXCR4* expression in sh*SDC2* tumours, albeit not significant **(**Figure 3B **)**. TGFβ has been shown to regulate the chemokine (C‐X‐C motif) ligand 12 (CXCL12) receptor (CXCR4) axis in TASCs, and this pathway contributes to the migratory properties of TASCs.^41^, ^42^ Using the same model the effect of TASCs transduced with an adenovirus containing *SDC2* (Ad*SDC2*) on tumour growth was compared to that of adenovirus control (AdControl)‐transduced TASCs. Increased levels of *SDC2* RNA in Ad*SDC2*‐transduced TASCs were ensured before transplantation (Figure 3C), and flow cytometry analysis was used to ensure transduced cells continued to express stromal cell markers. AdCon and Ad*SDC2*‐transduced cells expressed stromal cell markers CD90, CD105 and CD73 and were negative for haematopoietic markers CD45 and CD34, monocyte/macrophage markers CD14 and CD11b, and lack MHC‐II expression (Supplementary Figure 2). Overexpression of *SDC2* in TASCs causes a significant increase in breast tumour growth at Day 17 after initial detection of palpable tumour growth (*P* < .01) (Figure 3C). Tumours containing Ad*SDC2*‐transduced TASCs had elevated RNA levels of *SDC2*, *SMAD7* and *PAI‐1* (*P* < .05) (Figure 3D **)**. Ad*SDC2*‐transduced TASCs also enhanced lung metastasis of MDA‐MB‐231 cells—with increased numbers of metastatic nodules compared to those that received AdControl‐transduced TASCs (Figure 3E and Supplementary Figure 3). These in vivo studies demonstrate that stromal‐derived *SDC2* regulates primary tumour growth and metastasis and modulates TGFβ signalling within the TME, suggesting that blocking stromal‐derived syndecan‐2 has the potential to inhibit tumour growth and metastasis.

### 3.4 Generation of a Syndecan‐2‐peptide that inhibits TGFβ signalling and possesses antimigratory properties

**FIGURE 4:**
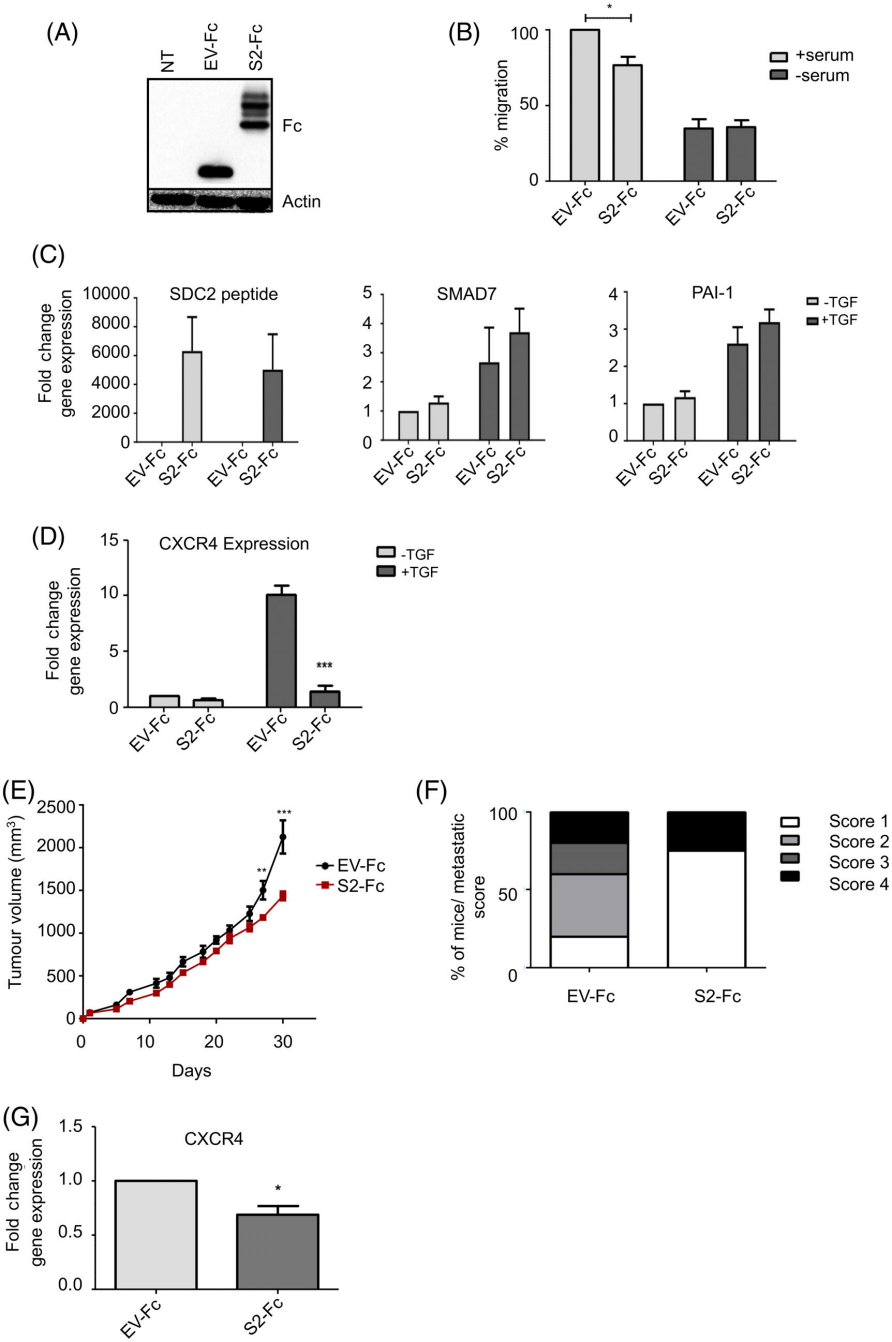
Syndecan‐2 peptide decreases breast tumour growth and TGFβ signalling. A, Western blot analysis demonstrating overexpression of Fc‐tagged syndecan‐2 peptide (S2‐Fc) and Fc‐empty vector control (EV‐Fc) in TASCs compared to nontransfected TASCs (NT). B, The xCelligence system was used to determine the ability of EV‐Fc‐ and S2‐Fc‐transfected TASCs to migrate towards serum‐containing media. Levels of migration were normalised to the positive control (ie, EV‐Fc + serum). C, EV‐Fc or S2‐Fc expressing TASCs were treated with TGFβ, RNA was prepared and levels of TGFβ‐regulated genes were determined by RT‐qPCR, n = 4. D, EV‐Fc or S2‐Fc expressing TASCs were treated with TGFβ_,_ following treatment RNA was prepared and levels of *CXCR4* were determined by RT‐qPCR. E, NOD:SCID tumours containing TASCs expressing S2‐Fc show a significant decrease in tumour growth compared EV‐Fc control tumours (n = 5 [EV‐Fc] n = 4 [S2‐Fc]). F, Lungs were removed and examined for metastatic nodules by H&E staining. The bar graph indicates the metastatic score/lung from the two experimental groups. G, Tumours were excised and RT‐qPCR was performed to determine the levels of *CXCR4* expression. *P ≤ .05; **P ≤ .01; ***P ≤ .001 [Color figure can be viewed at wileyonlinelibrary.com]

Therefore, to develop a potential blocking agent for syndecan‐2 biological activity, a *SDC2* peptide fragment encompassing amino acid 1‐87 was cloned into the pFUSE‐human‐IgG1‐Fc1 vector (InvivoGen) to stabilise expression.^43^ Overexpression of syndecan‐2‐peptide (Figure 4A) did not affect proliferation rates (Supplementary Figure 4A **)** or cell viabilty (Supplementary Figure 4B **)** of TASCs; however, it did inhibit the migratory properties of TASCs compared to empty vector control transfected TASCs (Figure 4B). To ascertain if *SDC2*‐peptide affected TGFβ signalling, TASCs were treated with TGFβ and expression of TGFβ regulated genes determined. There was no difference in TGFβ‐induced upregulation of *SMAD7* and *PAI‐1* observed between *SDC2*‐peptide‐ and control‐transfected TASCs **(**Figure 4C **)**. However, TGFβ‐induced upregulation of *CXCR4* was strongly inhibited in *SDC2*‐peptide expressing TASCs (Figure 4D). Hence similar to *SDC2* knockdown, *SDC2*‐peptide inhibits TGFβ signalling and the migratory potential of TASCs.

To determine the effect of manipulating stromal syndecan‐2 with syndecan‐2‐peptide within the TME, the orthotopic breast cancer model described previously was used. Growth rates were compared for tumours generated with human patient‐derived TASCs overexpressing *SDC2*‐peptide (S2‐Fc) and those generated with control‐transfected (EV‐Fc) TASCs. Overexpression of *SDC2*‐peptide in TASCs resulted in significantly lower tumour volume by Day 27 compared to tumours containing control‐transfected TASCs (Figure 4E) and lower metastatic scores in the lungs (Figure 4F). Tumours initiated with TASCs overexpressing *SDC2*‐peptide also had lower expression of *CXCR4* (Figure 4G).

### 3.5 Syndecan‐2‐peptide reduces the immunosuppressive properties of TASCs enabling activation of T cells

**FIGURE 5:**
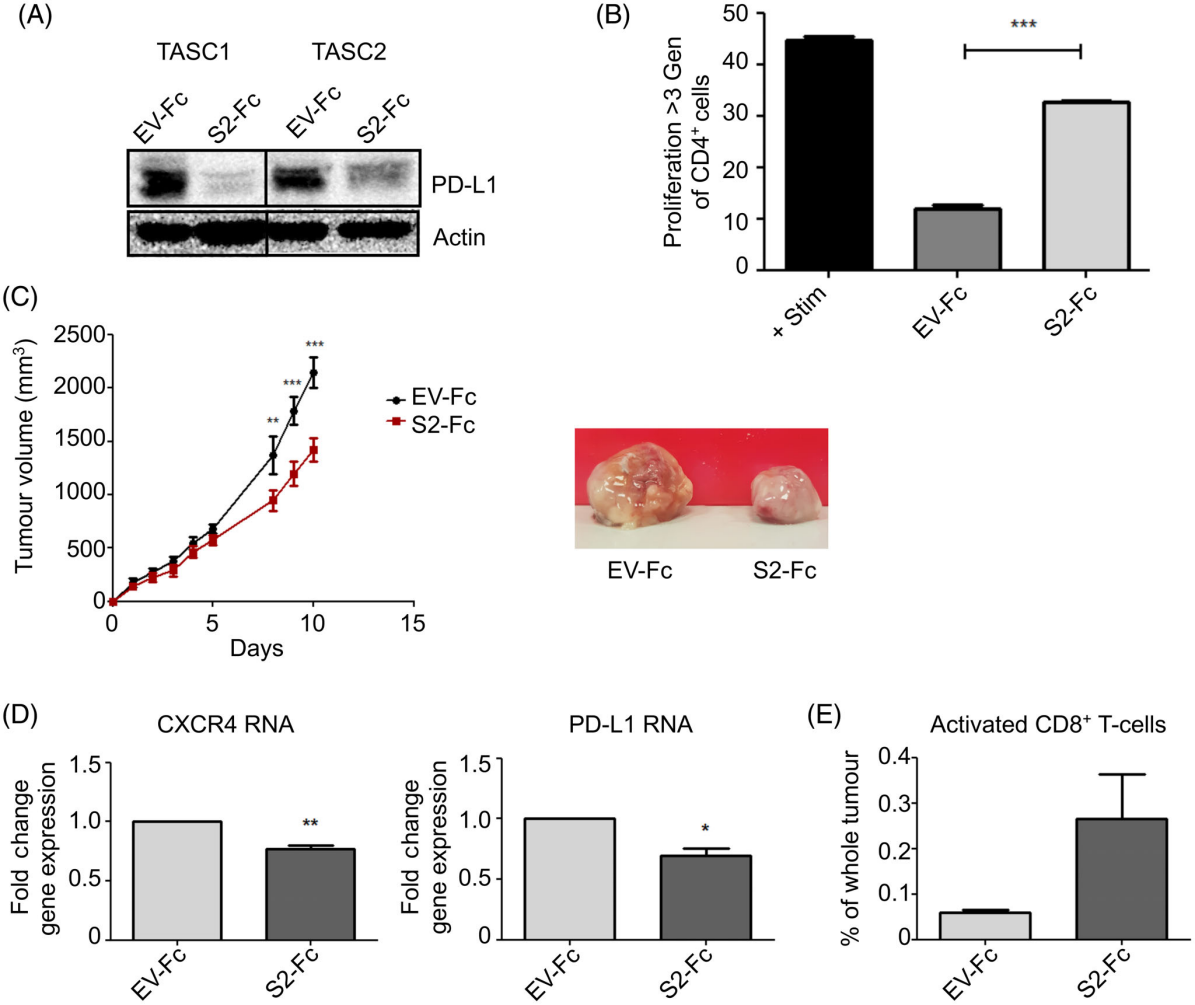
Syndecan‐2 peptide reduces the immunosuppressive properties of TASCs, reduces tumour growth and increases the percentage of activated T cells within the TME. A, TASCs were transfected with EV‐Fc control or S2‐Fc, 48 hours later protein extracts were prepared and *PD‐L1* levels were determined by Western blot analysis. B, EV‐Fc or S2‐Fc expressing TASCs were cocultured with CD3/CD28‐activated peripheral blood mononuclear cells (PBMCs) at a ratio of 1:10. Flow cytometry of CFSE‐labelled CD4^+^ T cells was used to measure the level of proliferation. Data were compared by one‐way analysis of variance with Tukey's multiple comparison posttest. n = 3. C, EO771 tumours containing TASCs expressing S2‐Fc show a significant decrease in tumour growth compared EV‐Fc control tumours (n = 4 [EV‐Fc] n = 4 [F2‐Fc]). D, Tumours were excised and RT‐qPCR was performed to determine the levels of *CXCR4* and *PD‐L1*. E, Flow cytometry analysis of tumours shows trends towards an increase in the number of CD8^+^, CD4^−^, CD62L^lo^, CD44^hi^ and CD25^+^‐activated T cells. *P ≤ .05; **P ≤ .01; ***P ≤ .001 [Color figure can be viewed at wileyonlinelibrary.com]

In addition to regulating TASC migration, CXCR4 contributes to the immunosuppressive properties of TASCs.^19^ Additionally, PD‐L1 is a TGFβ‐regulated gene that controls the immunosuppressive properties of TASCs.^10^, ^44^ Therefore, we wanted to establish if similar to *SDC2* knockdown, syndecan‐2‐peptide decreased the immunosuppressive properties of TASCs. Figure 5A shows that TASCs expressing syndecan‐2‐peptide expressed lower levels of PD‐L1 compared to control expressing cells. Taken these data together, it would be suggested that syndecan‐2‐peptide causes a decrease in *CXCR4* and PD‐L1 expression, which has the potential to inhibit the immunosuppressive of TASCs. Indeed, in T‐cell stimulation assays, TASCs expressing *SDC2*‐peptide were less efficient at inhibiting CD4^+^ T‐cell proliferation compared to control‐transfected‐TASCs (26 ± 1% inhibition (*SDC2*‐peptide) compared to 73 ± 2% inhibition (EV‐control)). **(**Figure 5B **)**. Importantly, empty vector‐control expressing TASCs suppressed T‐cell proliferation to similar levels as non‐transfected TASCs observed in Figure 2E (73 ± 2% vs 69.1 ± 2.3%, respectively). Thus, induced expression of syndecan‐2‐peptide results in inhibition of TGFβ‐induced gene products and loss of T‐cell inhibitory effects of TASCs.

To establish if syndecan‐2‐peptide affected the immunosuppressive properties of TASCs within the TME, we developed a syngeneic immune‐competent orthotopic model whereby EO771 mouse BCCs were coimplanted with mouse TASCs into the mammary fat pad of C57BL/6 female mice at a ratio of 10:1. TASCs were transfected with syndecan‐2‐peptide or empty vector control. Figure 5C shows that overexpression of syndecan‐2‐peptide in TASCs resulted in significantly lower tumour volume by Day 9 compared to control tumours. Tumours containing TASCs overexpressing syndecan‐2‐peptide also had lower levels of *CXCR4* and *PD‐L1* expression (Figure 5D), indicating a less immunosuppressive environment. Hence, we looked at levels of specific immune cell populations within the TME. There was no difference in the percentage of CD4^+^ cells, neutrophils, dendritic cells, monocytes or macrophages between syndecan‐2‐peptide expressing and control tumours (Supplementary Figure 5). However, there was an observed increase in the percentage of CD8^+^, CD4^−^, CD62L^lo^, CD44^mid^ and CD25^−^‐activated T cells within the TME of syndecan‐2‐peptide expressing tumours compared to control tumours (Figure 5E).

## 4 DISCUSSION

In our study, we demonstrate that syndecan‐2 is expressed on the cell surface of TASCs within human and mouse breast tumours. TASCs play a significant role in promoting tumour growth, metastasis and immunosuppression within the TME; hence, we wanted to unravel the function of syndecan‐2 in TASCs. Our data indicate that syndecan‐2 is important for TGFβ signalling in TASCs and regulates TGFβ‐responsive genes *Smad7*, *CTGF*, *PAI‐1* and *CXCR4*. TGFβ signalling within the TME contributes to tumour growth and metastasis, and elevated expression of stromal TGFβ in breast, colorectal and prostate cancer is associated with poor prognosis and locally advanced disease.^45^ Therefore, modulation of TGFβ signalling within the stromal compartment of tumours has the potential to alleviate tumourigenesis. Indeed, our data indicate limiting syndecan‐2 (by *SDC2* knockdown or syndecan‐2 peptide overexpression) in TASCs attenuates TGFβ signalling within the breast TME reducing tumour growth and metastasis. On the other hand, overexpression of *SDC2* in TASCs increases TGFβ signalling within human xenograft tumours, enhancing tumour growth and metastasis. This is the first study to show TASC‐derived syndecan‐2 controls breast tumourigenesis via TGFβ signalling. Previous studies demonstrate that syndecan‐2 inhibition (via *SDC2* knockdown or syndecan‐2‐peptide) within the epithelial compartment of tumours also reduces tumour growth and metasatasis in immune‐compromised models.^31^, ^46^ Therefore, in future studies, administration of exogenous Fc‐tagged syndecan‐2 peptide has the potential to be more stable in vivo, inhibit both stromal‐ and epithelial‐derived syndecan‐2 and thus be more efficacious at inhibiting tumourigenesis.

TGFβ signalling also contributes to the immunosuppressive properties of TASCs.^17^ Our study demonstrates that reducing syndecan‐2 activity (by *SDC2* knockdown or syndecan‐2‐peptide overexpression) in TASCs inhibits TGFβ‐induced immunosuppressive genes, *PD‐L1* and *CXCR4*, and this correlates with a reduction in the ability of TASCs to suppress T‐cell proliferation in vitro. Concordantly, the syndecan‐2‐peptide‐induced reduction in *CXCR4* and *PD‐L1* expression in TASCs coincided with an increase in the levels of activated T cells within the TME and a decrease in tumour growth in an immune competent syngeneic breast cancer model. This correlates with previous studies that show dual inhibition of CXCR4‐CXCL12 (AMD3100) and PD‐1‐PD‐L1 (PD‐1 antibody) pathways reduce immunosuppression within ovarian tumours allowing the recruitment of effector T cells and a reduction in tumour growth.^47^ In other studies, blocking the CXCL12/CXCR4 axis (AMD3100) in the stromal compartment of pancreatic and breast tumours reactivates T‐cell cytotoxic capacity within the TME, thereby rendering these tumours susceptible to immune checkpoint inhibitor therapies (eg, α‐PD‐L1, α‐PD‐1 and α‐CTLA‐4).^19^, ^48^ This would imply due to the ability of syndecan‐2 peptide to reduce stromal *CXCR4* and *PD‐L1* expression, this gives syndecan‐2 peptide the potential to render breast tumours more susceptible to cancer immunotherapies such as α‐CTLA‐4 antibodies, tumour‐infiltrating lymphocytes (TILs) and/or chimeric‐antigen receptor T cells (CAR T cells).

In summary, our data indicate that syndecan‐2 is present in the stromal compartment of breast tumours and contribute to the oncogenic properties of TASCs by promoting TGFβ signalling, tumour growth, metastasis and immunosuppression. This study unravels the potential of syndecan‐2‐peptide as an antitumourigenic agent, exerting a novel multi‐modal attenuation of TGFβ signalling, limiting metastasis and reducing tumour growth by relieving immunosuppression mediated by TASCs within the TME.

## CONFLICT OF INTEREST

TOB is founder, director and shareholder of Orbsen Therapeutics Ltd. SJE, PL, LW LMD and SA are employees and shareholders of Orbsen Therapeutics Ltd. LOF is a former employee and shareholder of Orbsen Therapeutics Ltd. MG, MK, RMD, ECR and LRB have no conflicts of interest to declare.

## ETHICS STATEMENT

Ethical approval was obtained from the research ethics committee at the National University of Ireland, Galway. After written informed consent, fresh specimens of human breast tumours were harvested from patients undergoing surgery at University College Hospital Galway.

## Supporting information


**Appendix** **S1:** Supplementary Information

## Data Availability

The data that support the findings of this study are available from the corresponding author upon reasonable request
