# Supplementary material for: Targeting stromal cell Syndecan‐2 reduces breast tumour growth, metastasis and limits immune evasion
Source: Int J Cancer. 2020 Dec 2;148(5):1245–59. doi: 10.1002/ijc.33383 (PMC7839764; doi:10.1002/ijc.33383)
Supplement: Supplementary file 1 — Appendix S1: Supplementary Information [file IJC-148-1245-s001.pdf]

TITLE PAGE

Targeting stromal cell Syndecan-2 reduces breast tumour growth, metastasis and limits immune evasion.

Running title: Syndecan-2^{+}-stromal cells enhance breast tumourigenesis

Paul Loftus[1,2], Luke Watson[1], Laura M Deedigan[2], Eva Camarillo-Retamosa[2], Róisín M Dwyer[1], Lisa O'Flynn[2], Matthew Griffin[1], Timothy O'Brien[1], Michael Kerin[1], Stephen J Elliman[2], Laura R Barkley[1*].

Table of contents

Supplementary Figure 1
Supplementary Figure 2
Supplementary Figure 3
Supplementary Figure 4
Supplementary Figure 5

Supplementary Figure 1

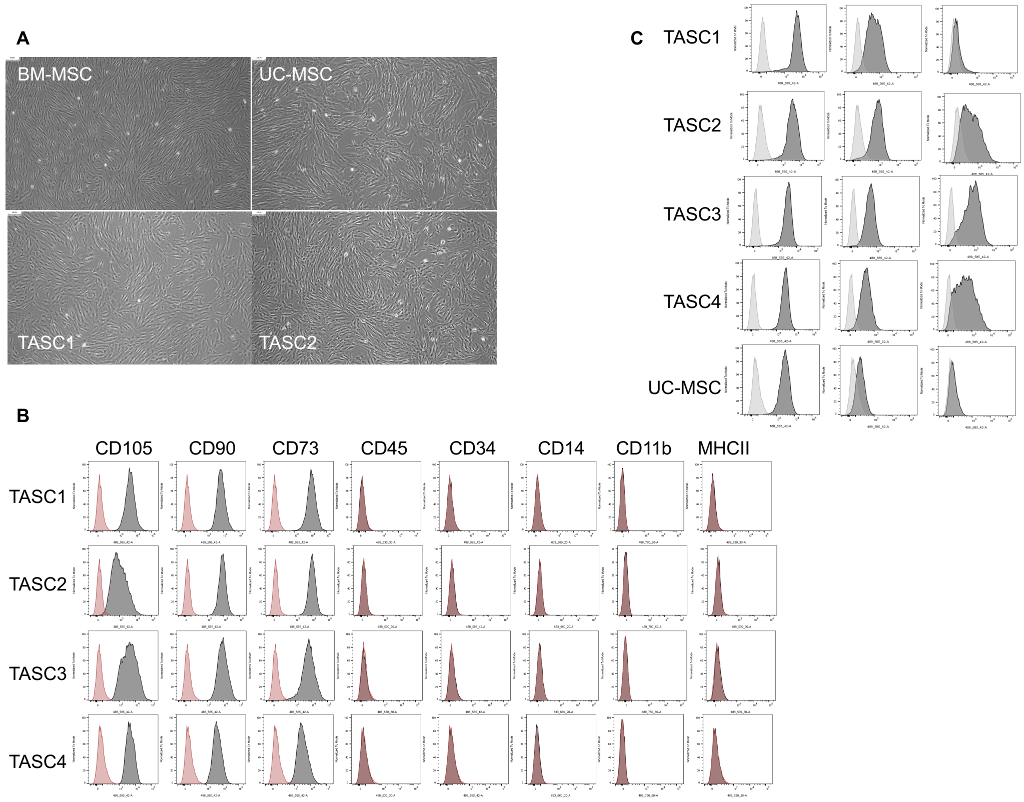

Supplementary Figure 1. Comparison between TASCs and normal MSCs. (A) Morphological comparison of TASCs to normal bone marrow (BM-MSC) and umbilical cord-derived (UC-MSC) MSCs at passage 2. (B) TASCs were isolated, cultured and expanded from human breast cancer tissues. Flow cytometry analysis confirmed that cells expressed CD90, CD105, CD73, and were negative for CD45, CD34, CD14, CD11b, and MHC-II expression. (C) Flow cytometry analysis confirmed that TASCs from 4 different donors expressed NG2, PDGFRα and gp38.

Supplementary Figure 2

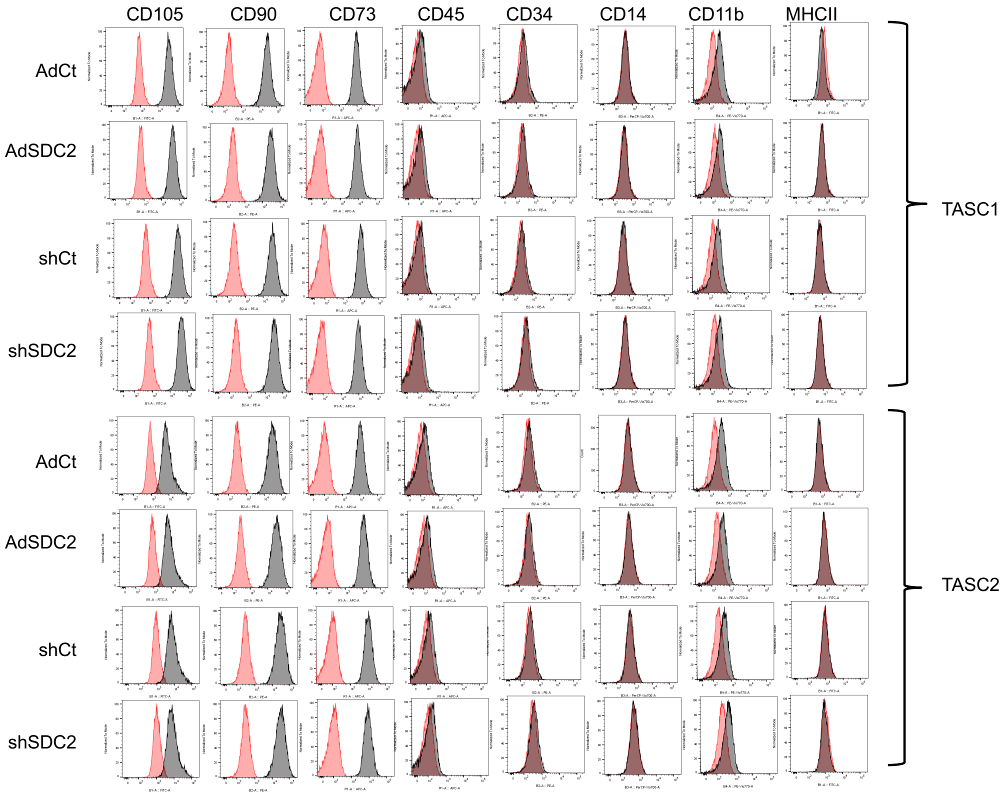

Supplementary Figure 2. Adenovirus transduction does not effect cell surface expression of TASCs. TASCs were transduced with adenovirus control (AdCt), adenovirus SDC2 (AdSDC2), adenovirus shRNA control (shCt) or adenovirus shRNA-SDC2 (shSDC2) for 48 hours. Flow cytometry analysis confirmed that cells still expressed CD90, CD105, CD73, and were negative for CD45, CD34, CD14, CD11b, and MHC-II expression.

Supplementary Figure 3

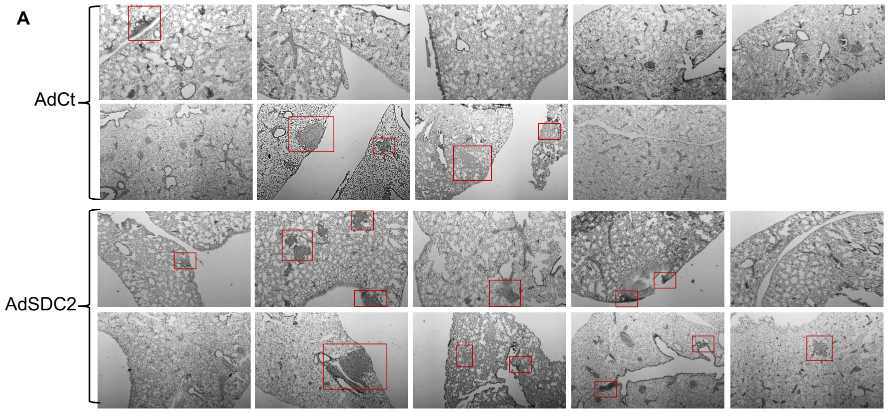

Supplementary Figure 3. Overexpression of stromal SDC2 increases metastasis to the lung in a xenograft breast cancer model. (A) NOD:SCID mice were injected with TASCs:MDA-MB-231 into the mamary fat pad and approximately 12 weeks later lungs were removed and examined for metastatic nodules by H&E staining. Representative images of H&E stained lungs of AdCt (n=9) and AdSDC2 (n=10) mice. Red boxes indicate metastatic lesions.

Supplementary Figure 4

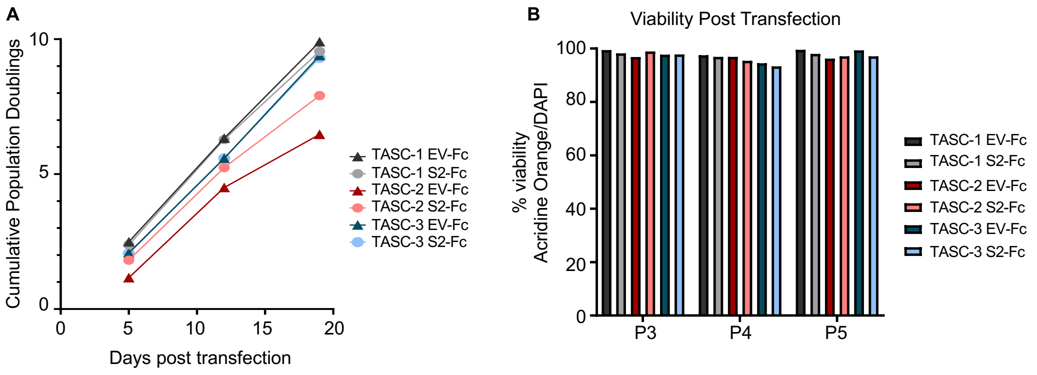

Supplementary Figure 4. Overexpression of syndecan-2-peptide does not effect proliferation or viability of TASCs. (A) Cumulative population doublings after transfection of TASCs with empty vector Fc contol (EV-Fc) or Syndecan-2-Fc peptide (S2-Fc) were measured. (B) Viability of EV-Fc or S2-Fc transfected TASCs were determined by Acridine orange/DAPI co-stain and analysed by Chemometec Nucleocounter® NC-200.

Supplementary Figure 5

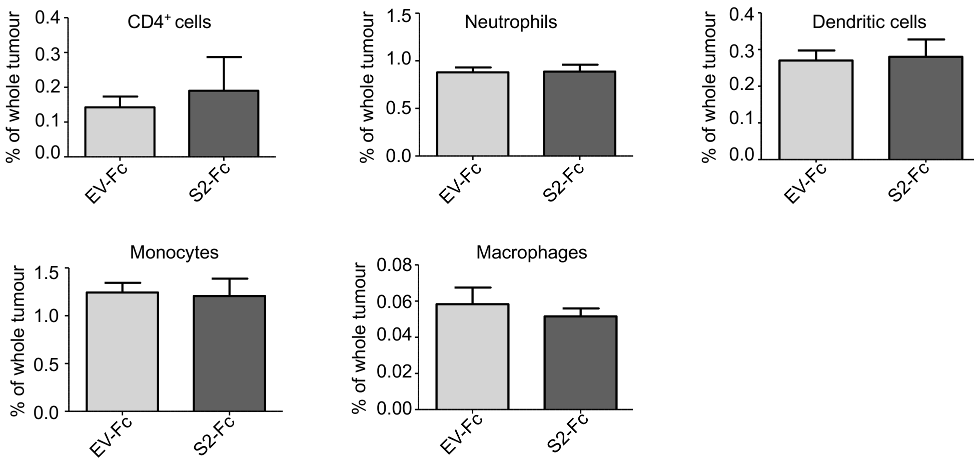

Supplementary Figure 5. Effect of TASC-SDC2-peptide expression on immune cell content within the TME. EO771 tumours containing TASCs expressing S2-Fc or EV-Fc were excised and flow cytometry analysis of tumours was perfomed to determine the percentage of $CD4^+$ T cells, neutrophils ($CD11b^+$ $Ly6G^+$), dendritic cells ($Ly6G^-$ $CD11b^-$ $CD11c^+$), monocytes ($Ly6G^-$ $CD11b^+$ $CD11c^-$ $Ly6C^{hi}$ $F4/80^{mid\text{-}low}$) and macrophages ($Ly6G^-$ $CD11b^+$ $CD11c^-$ $Ly6C^{lo}$, $F4/80^+$) within EV-Fc and S2-Fc expressing tumours.
